# Dissecting the Prognostic Significance and Functional Role of Progranulin in Chronic Lymphocytic Leukemia

**DOI:** 10.3390/cancers11060822

**Published:** 2019-06-13

**Authors:** Lena Schulze-Edinghausen, Claudia Dürr, Selcen Öztürk, Manuela Zucknick, Axel Benner, Verena Kalter, Sibylle Ohl, Viola Close, Patrick Wuchter, Stephan Stilgenbauer, Peter Lichter, Martina Seiffert

**Affiliations:** 1 Division of Molecular Genetics, German Cancer Research Center (DKFZ), 69120 Heidelberg, Germany; claudi.duerr@gmail.com (C.D.); s.oeztuerk@dkfz-heidelberg.de (S.Ö.); v.kalter@dkfz-heidelberg.de (V.K.); s.ohl@dkfz-heidelberg.de (S.O.); peter.lichter@dkfz-heidelberg.de (P.L.); 2 Oslo Centre for Biostatistics and Epidemiology, Department of Biostatistics, Institute of Basic Medical Sciences, Faculty of Medicine, University of Oslo, 0372 Oslo, Norway; manuela.zucknick@medisin.uio.no; 3 Division of Biostatistics, German Cancer Research Center (DKFZ), 69120 Heidelberg, Germany; benner@dkfz-heidelberg.de; 4 Internal Medicine III, University of Ulm, 89081 Ulm, Germany; viola.close@outlook.com; 5 Cooperation Unit Mechanisms of Leukemogenesis, German Cancer Research Center (DKFZ), 69120 Heidelberg, Germany; 6 Institute of Transfusion Medicine and Immunology, Medical Faculty Mannheim, Heidelberg University, German Red Cross Blood Service Baden–Württemberg–Hessen, 68167 Mannheim, Germany; patrick.wuchter@medma.uni-heidelberg.de; 7 Internal Medicine III, University of Ulm, 89081 Ulm, Germany; stephan.stilgenbauer@uniklinik-ulm.de; 8 Department of Internal Medicine I, Saarland University, 66421 Homburg, Germany; 9 German Cancer Research Consortium (DKTK), German Cancer Research Center (DKFZ), 69120 Heidelberg, Germany

**Keywords:** chronic lymphocytic leukemia, tumor microenvironment, progranulin, prognostic serum marker, cancer-associated fibroblasts

## Abstract

Chronic lymphocytic leukemia (CLL) is known for its strong dependency on the tumor microenvironment. We found progranulin (GRN), a protein that has been linked to inflammation and cancer, to be upregulated in the serum of CLL patients compared to healthy controls, and increased GRN levels to be associated with an increased hazard for disease progression and death. This raised the question of whether GRN is a functional driver of CLL. We observed that recombinant GRN did not directly affect viability, activation, or proliferation of primary CLL cells in vitro. However, GRN secretion was induced in co-cultures of CLL cells with stromal cells that enhanced CLL cell survival. Gene expression profiling and protein analyses revealed that primary mesenchymal stromal cells (MSCs) in co-culture with CLL cells acquire a cancer-associated fibroblast-like phenotype. Despite its upregulation in the co-cultures, GRN treatment of MSCs did not mimic this effect. To test the relevance of GRN for *CLL* in vivo, we made use of the Eμ-*TCL1* CLL mouse model. As we detected strong GRN expression in myeloid cells, we performed adoptive transfer of Eμ-*TCL1* leukemia cells to bone marrow chimeric *Grn*^−/−^ mice that lack GRN in hematopoietic cells. Thereby, we observed that CLL-like disease developed comparable in *Grn*^−/−^ chimeras and respective control mice. In conclusion, serum GRN is found to be strongly upregulated in CLL, which indicates potential use as a prognostic marker, but there is no evidence that elevated GRN functionally drives the disease.

## 1. Introduction

Chronic lymphocytic leukemia (CLL) is characterized by the outgrowth of malignant CD5^+^ B cells and their increased detection in patients’ blood [1]. Common clinical staging systems according to Rai or Binet reflect further tissue involvement, which becomes apparent by lymphadenopathy, and disruption of normal hematopoiesis in the bone marrow causing anemia and thrombocytopenia [2,3,4]. Despite recent advances in therapies targeting B cell receptor pathway-kinases, treatment resistance and aggressive cases remain major challenges impeding clinical control of CLL [5,6,7,8].

In contrast to chronic myeloid leukemia, CLL is not driven by a single deregulated pathway but diverse somatic mutations that affect various cellular processes have been detected in relatively low frequencies each [9,10,11]. It is widely accepted, that in addition to cell-intrinsic features the tumor microenvironment plays a substantial role in CLL pathogenesis. Kinetic studies revealed, that fractions of proliferating CLL cells particularly locate to tissue compartments where they interact with diverse accessory cells [12,13,14]. In addition, the crucial relevance of the CLL microenvironment is demonstrated by high apoptosis rates of CLL cells when they are cultured in vitro, which can be prevented by co-cultures with myeloid or non-hematopoietic stromal cells mimicking microenvironmental niches [15,16,17,18,19,20]. In the reciprocal crosstalk between CLL cells and their microenvironment, leukemia development is accompanied by the skewing of accessory cells, such as myeloid cells [21,22,23,24] or T cells [25,26,27,28,29,30], towards pro-tumorigenic phenotypes and compositions. Targeting myeloid cells [21,23] or modulating T cell functions by checkpoint inhibitors [31,32] showed promising efficacy against CLL in preclinical studies. Immunotherapeutic approaches are thus expected to complement future treatment strategies, and understanding of the crosstalk between CLL and accessory cells has the potential to reveal new therapeutic targets.

Not only immune cells but also non-hematopoietic stromal cells are increasingly recognized as functionally important components of the tumor microenvironment in many cancers. Cancer-associated fibroblasts (CAFs) can harbor pro-tumorigenic functions, for example by supporting cancer cell invasion or by inducing tumor-promoting immune modulation [33,34,35,36,37,38]. Mesenchymal stromal cells (MSCs) have been shown to be one origin of CAFs [39,40,41,42] and are of particular interest in CLL due to their abundance in the bone marrow, which builds a prognostically significant tissue niche for infiltrating CLL cells [43,44]. Although aspects of CLL-specific MSCs have been analyzed by transcriptome profiling in xenogeneic co-cultures [45] or after exposure to CLL-derived extracellular vesicles [46], a comprehensive characterization of CLL-associated, human MSCs was, to our knowledge, not performed so far.

Progranulin (GRN) is a secreted, cysteine-rich, 88 kDa large glycoprotein synonymous to acrogranin, epithelin precursor, and PC cell-derived growth factor [47,48,49,50,51,52]. GRN deficiency causes neurodegenerative diseases, namely frontotemporal dementia and neuronal ceroid lipofuscinosis [53,54,55,56]. Increased GRN serum or tissue levels, in contrast, were associated with cancer and connected to a negative prognosis, for example in carcinomas of the breast [57,58,59], ovary [60], liver [61], prostate [62], and lung [63], and in brain tumors like glioblastoma [64]. Göbel et al. have identified GRN as a novel negative prognostic factor in CLL in a study including mainly low clinically staged patients (Binet A) [65]. GRN was demonstrated to have growth factor activity and positively impacted on cancer growth in preclinical solid tumor models [52,66,67,68,69,70]. In addition, GRN was reported to fulfil versatile immune regulatory roles affecting macrophages [71,72,73,74,75,76], neutrophils [77,78], and T cells [79,80]. Of interest, GRN was shown to induce the differentiation of CAFs from resident fibroblasts in breast and pancreatic cancer [57,81]. Thus, GRN was a promising candidate to be tested as a potential driver of CLL and mediator within its microenvironment.

This study aimed at dissecting the role of GRN in CLL. In summary, serum GRN was validated to be strongly upregulated in CLL and associated with an increased risk for disease progression and death, but GRN did not affect CLL cells or CLL accessory cells in vitro, and there was no evidence that it functionally drives the disease in vivo.

## 2. Results

### 2.1. Progranulin is Strongly Upregulated in the Serum of CLL Patients and Associated with an Increased Risk of Disease Progression and Death

**Figure 1:**
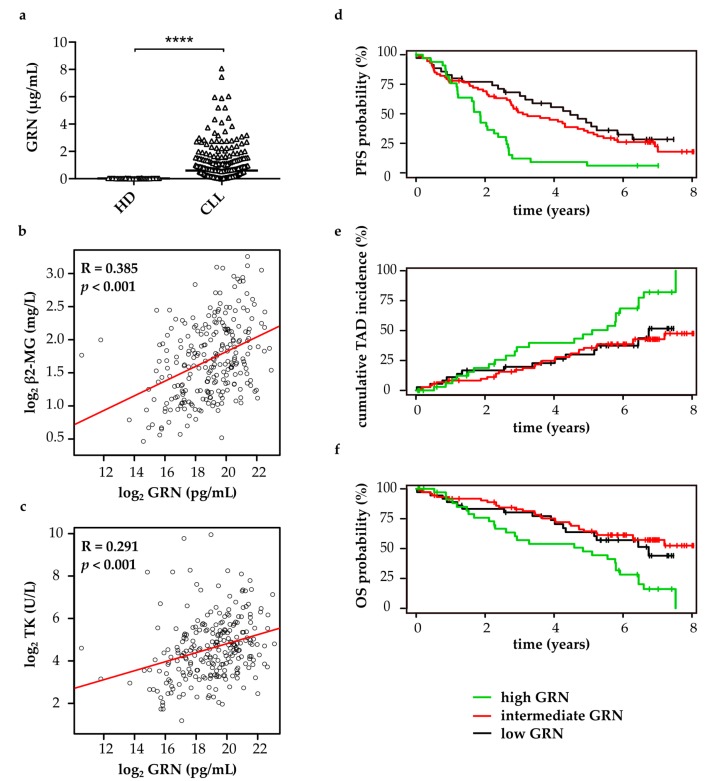
Elevated serum progranulin (GRN) is of negative prognostic relevance in CLL. (**a**) GRN serum levels in 249 CLL patients and 42 age- and sex-matched healthy donors (HD) were quantified by cytometric bead arrays. Individual data points and their median are plotted. Two-sided Mann-Whitney U test, *p* < 0.0001; (**b**,**c**) Correlations of GRN levels with (**b**) β2-microglobulin (β2-MG) or (**c**) thymidine kinase (TK) levels in the serum of 249 CLL patients, Pearson’s correlation coefficients (R), and *p* values are shown. Data were log_2_ transformed; (**d–f**) Association of GRN levels with (**d**) progression-free survival (PFS); (**e**) tumor-associated deaths (TAD), and (**f**) overall survival (OS) was analyzed by a univariate Cox hazard regression model. GRN levels were log_2_ transformed and entered as continuous variables. Results are depicted in Stone-Beran plots with estimates for high, intermediate and low GRN concentration shown in separate colors as indicated.

To evaluate the prognostic significance of progranulin (GRN) in CLL patients with advanced stage of disease, GRN was quantified in the serum of 249 patients included in the CLL8 study cohort (ClinicalTrials.gov: NCT00281918) and 42 age- and sex-matched healthy controls. All patients in the CLL8 cohort were in Binet stage B or C, treatment-naive, and required clinical intervention (patient characteristics summary in Supplementary Table S1). GRN serum levels were found statistically significantly elevated in CLL patients (median 605.60 ng/mL, range 1.51 ng/mL–8.07 µg/mL) compared to healthy controls (median 25.81 ng/mL, range 2.39 ng/Ml–60.84 ng/mL) (Figure 1a). Furthermore, GRN concentrations showed a weak positive correlation with β2-microglobulin (Figure 1b) and thymidine kinase (Figure 1c) serum levels, which represent established negative prognostic markers in CLL [82]. Of note, high GRN was statistically significantly associated with shorter progression-free survival (Figure 1d), increased tumor-associated deaths (Figure 1e), and shorter overall survival (Figure 1f) of the patients (Supplementary Table S2). Multivariate analyses revealed that the negative prognostic value of GRN was not independent of the covariates rituximab treatment, age, 11q deletion, 17p deletion, and IGHV mutational status, the latter four being of negative prognostic value in CLL, in the CLL8 cohort (Supplementary Table S2). This suggested that at least one of these covariates might be confounded with GRN.

### 2.2. Progranulin does not Directly Support CLL Cells In Vitro

**Figure 2:**
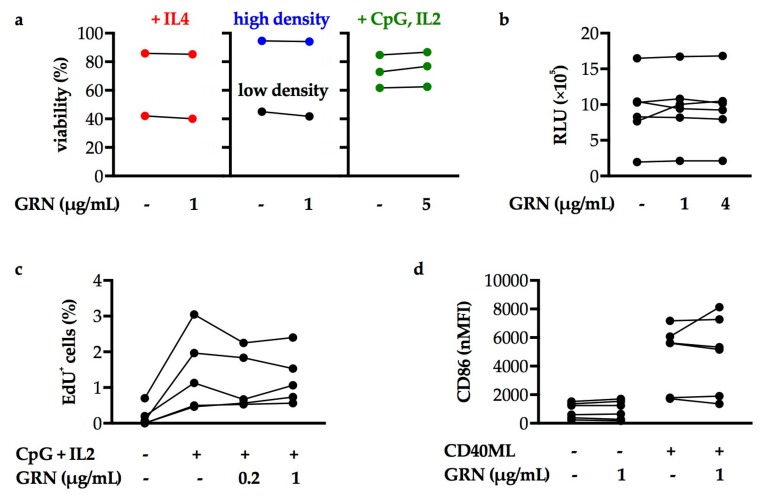
CLL cells cultured in vitro are not directly affected by treatment with recombinant GRN. Data points in plots depict means of 2–5 technical replicates. The number of data points thus indicates biological replicates (n). Wilcoxon matched-pairs signed rank tests of samples with or without GRN treatment, all *p* values > 0.05. (**a**) Percentage of viable CLL cells in the indicated culturing conditions and treated with the indicated concentrations of GRN after 2 days (in low cell density (n = 1) or with 50 units/mL IL4 (n = 2)), 4 days (with 2 µM CpG oligodeoxynucleotides and 100 U/mL IL2 (n = 3)), or 9 days (in high cell density, n = 1) of culture was assessed by Annexin V/7AAD apoptosis assay via flow cytometry. (**b**) CLL cell viability was assessed by CellTiter-Glo assay after 2 days of incubation with the indicated concentrations of GRN (intermediate cell density, no additional factors added). RLU: relative luminescence unit. n = 6 biological replicates. (**c**) The fraction of proliferating CLL cells represented by EdU^+^ cells was assessed by Click-iT assay after treatment with 2 μM CpG and 100 U/mL IL2. n = 5 biological replicates. (**d**) CLL cell activation was measured by CD86 surface levels via flow cytometry after treatment with 100 ng/mL CD40 mega-ligand (CD40ML). 50 U/mL IL4 was added in all conditions. nMFI: normalized median fluorescence intensity (MFI CD86−MFI IgG isotype control). n = 6 biological replicates.

As GRN was strongly upregulated and associated with increased risk of progression and death in CLL, we hypothesized that it could be a functional driver of the disease. Thus, the effect of recombinant GRN on primary, patient-derived CLL cells was tested in vitro (patient characteristics summarized in Supplementary Table S3). CLL cells were cultured under different conditions, such as in high cell density, or in growth medium supplemented with soluble factors, modeling aspects of microenvironmental niches and achieving enhanced cell survival, proliferation, and activation. Applied concentrations of GRN were based on the levels detected in CLL patients’ serum (see Figure 1a). GRN addition to these cultures did not alter the viability of CLL cells as determined by flow cytometric (Figure 2a) or luminescence assays (Figure 2b). To evaluate the effect of GRN on proliferation, CLL cells were stimulated with CpG oligodeoxynucleotides and IL2 as described previously, as CLL cells do not enter the cell cycle in vitro without these stimuli [83,84]. While this successfully induced proliferation of CLL cells, the addition of recombinant GRN did not significantly impact on the fraction of proliferating cells (Figure 2c). Finally, CLL cells were activated by CD40 mega-ligand (CD40ML), a synthetic protein of two linked dimeric CD40L imitating contact with T helper cells. While CD40ML increased CLL cellular CD86 expression, a marker for activation, their activation status was not significantly different upon GRN treatment, both in the absence and in presence of CD40ML (Figure 2d). In conclusion, there was no evidence that viability, proliferation and activation status of CLL cells are affected by GRN in vitro.

### 2.3. Progranulin does not Induce the Formation of CLL-Specific Cancer-Associated Fibroblasts (CAFs)

An alternative mechanism by which GRN could potentially drive CLL is by mediating the formation of a CLL-promoting microenvironment. Interestingly, we observed, that GRN secretion was induced in co-cultures of CLL cells with non-hematopoietic stromal cells (Supplementary Figure S1), which are well-known to support the survival of CLL cells in vitro [16,17,20,85]. GRN was previously shown to mediate the acquisition of cancer-associated fibroblast (CAF)-like phenotypes in resident fibroblasts in the context of breast and pancreatic carcinomas [57,81]. Our observations in combination with published data thus enforced the hypothesis, that GRN facilitates the induction of CLL-specific CAFs.

**Figure 3:**
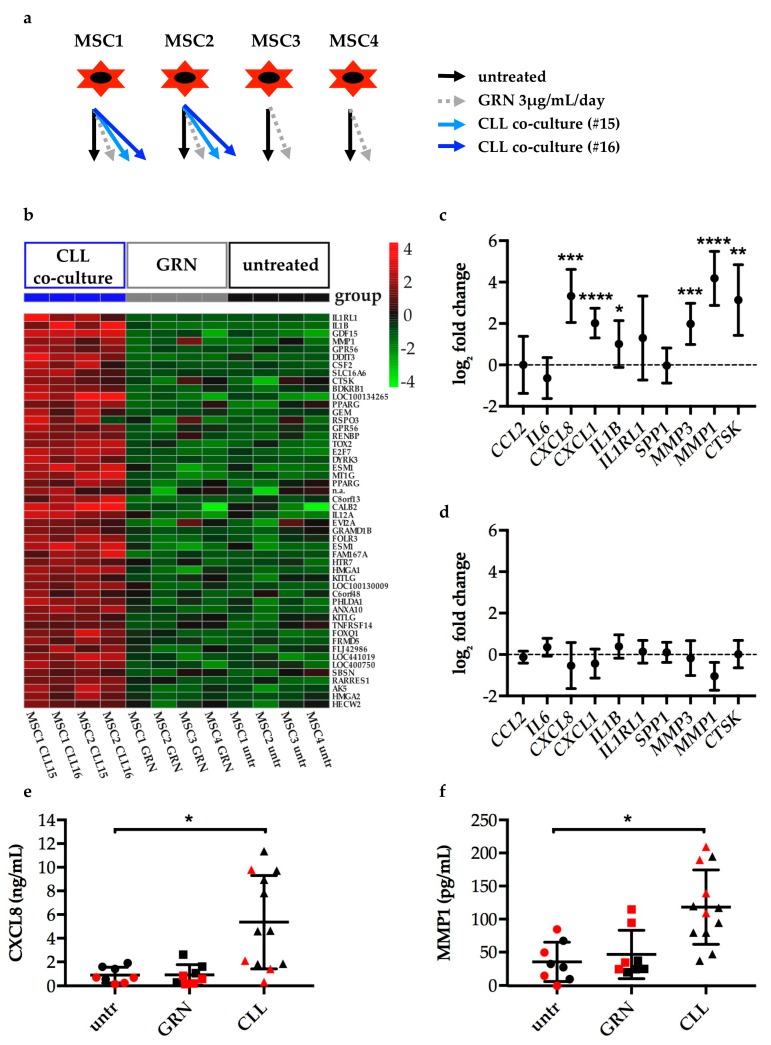
Mesenchymal stromal cells (MSCs) in co-culture with CLL cells acquire a cancer-associated fibroblast (CAF)-like phenotype which is not recapitulated by GRN treatment. (**a**) Scheme of 12 samples that were submitted for gene expression profiling (GEP) by Illumina BeadChip arrays. Human primary MSCs were either left untreated (black), treated with 3 µg/mL/day GRN (grey), or co-cultured with CD19-sorted CLL cells of two different patients (blue, #15 and #16, patient characteristics according to Supplementary Table S3) for 5 days. CLL cells were thoroughly removed from co-cultures before RNA isolation. (**b**) Heatmap of the top 50 upregulated genes in MSCs by CLL co-culture determined by Illumina BeadChip array analysis (listed in Supplementary Table S4). The plot shows mean-centered expression values for the respective Illumina probes of all samples including GRN treated ones. (**c**) Gene expression of MSCs co-cultured with CD19-sorted CLL cells for 3 days was determined by quantitative real-time PCR (qPCR). Data analysis was performed by ∆∆Ct method relative to the reference gene *VIM* and the untreated MSC control. Normalization to *ACTG1*, *RPS29*, and *FTL* showed similar results. Fold changes were log_2_ transformed. Plots show mean log_2_ fold change with SD, n = 8 different MSC donor and CLL donor combinations. One-sample t test was performed comparing log_2_ fold changes to 0. * *p* < 0.05, ** *p* < 0.01, *** *p* < 0.001, **** *p* < 0.0001. (**d**) Gene expression of MSCs treated with 3 µg/mL/day GRN for 3 days was determined by qPCR. Data display and analysis analogous to (**c**). n = 4 independent experiments using MSCs from 2 donors. All *p* values > 0.05. (**e**,**f**) CXCL8 or MMP1 in cell culture supernatants of samples analyzed by GEP (red data points) and qPCR shown in c and d (black data points) were quantified by cytometric bead array or ELISA, respectively. n = 8 (untreated and GRN treated MSCs); n = 12 (co-cultures). Means and SD are plotted. Wilcoxon matched-pairs signed rank tests were performed for untreated vs. GRN treated group (8 pairs, *p* values > 0.05) and untreated vs. CLL co-cultured group (6 pairs, using means of two technical replicates for CLL co-cultures, *p* = 0.0312 (MMP1), *p* = 0.0312 (CXCL8)).

To address this question, primary healthy donor bone marrow-derived mesenchymal stromal cells (MSCs) were either cultured alone, treated with recombinant GRN, or co-cultured with primary patient-derived CLL cells (experimental setup is illustrated by Figure 3a). Subsequently, gene expression of the MSCs was quantified by Illumina HumanHT-12 v4 Expression BeadChip arrays. Thereby, genes assigned to 454 Illumina probes were identified to be significantly altered by CLL co-culture compared to control MSCs cultured alone (top upregulated genes are listed in Supplementary Table S4, full data have been deposited in NCBI’s Gene Expression Omnibus [86], accession number GSE129108). Contrary, and arguing against the hypothesis, there were no significant transcriptional changes detected in GRN treated compared to untreated MSCs. More particular, GRN treated MSCs did not recapitulate the phenotypical changes induced in MSCs by CLL cell co-culture (Figure 3b).

Among the genes that were upregulated in MSCs upon CLL cell co-culture, we identified coding genes for several proteases, such as matrix metalloproteinase 1 (MMP1) or cathepsin k (CTSK), or inflammatory factors, such as interleukin 1 β (IL1B) or C-X-C motif chemokine ligand 8 (CXCL8). Our results were thus in line with features commonly associated with CAFs, like extracellular matrix remodeling and immune modulation [33,34,35,36,37,38]. To validate and re-evaluate the findings, 10 genes associated with these functions—and found upregulated in the here presented microarray-based gene expression profile of MSCs in CLL co-culture or in published CAF signatures [33,45]—were selected to be analyzed by quantitative real-time PCR (qPCR). This confirmed that CLL co-cultured MSCs upregulated IL1B, CXCL8, and CXCL1, as well as MMP1, MMP3, and CTSK (Figure 3c). On the other hand, none of the selected genes were significantly deregulated in MSCs by GRN treatment (Figure 3d). Finally, the data could be confirmed by quantification of the most deregulated genes as per qPCR in the cell culture supernatants on the protein level. Both CXCL8 (Figure 3e) and MMP1 (Figure 3f) were detected in increased amounts in the co-cultures but not in the GRN treated MSC cultures compared to untreated MSCs cultured alone.

Summarizing the findings, MSCs in co-culture with CLL cells acquired a CAF-like phenotype. Despite its upregulation in the co-cultures, GRN did not induce CLL-specific CAFs.

### 2.4. Progranulin does not Impact on Leukemia Development in Eμ-TCL1 Adoptive Transfer CLL Mouse Models

As no significant effect of GRN on CLL cells or accessory cells was detected in vitro, in vivo models were required to ultimately investigate whether GRN is of functional relevance in CLL. Therefore, the well-established Eμ-*TCL1* adoptive transfer CLL mouse model was used, in which immunocompetent syngeneic wild type mice develop a CLL-like disease a few weeks after they are transplanted with malignant splenocytes from a leukemic transgenic Eμ-*TCL1* mouse [87,88]. When Eμ-*TCL1* cells were adoptively transferred to syngeneic *Grn*^−/−^ mice and heterozygous control mice, only the latter developed leukemia over time (Supplementary Figure S2a). At the study endpoint 10 weeks after transplantation, leukemic cell load in blood, spleen, and peritoneal cavity (Supplementary Figure S2b), as well as the spleen weight (Supplementary Figure S2c), were significantly higher in the heterozygous control mice compared to the *Grn*^−/−^ mice. However, in follow-up studies on this experiment and Eμ-*TCL1* transplantations to other knockout mouse lines, we could recently provide evidence arguing that the observation of non-engraftment in *Grn*^−/−^ mice was most likely caused by a CD8^+^ T cell-mediated immune response of *Grn*^−/−^ mice against *Grn* expressing Eμ-*TCL1* leukemia cells [89]. The experimental setup was thus not suitable to draw reliable conclusions on the functional relevance of GRN in CLL.

**Figure 4:**
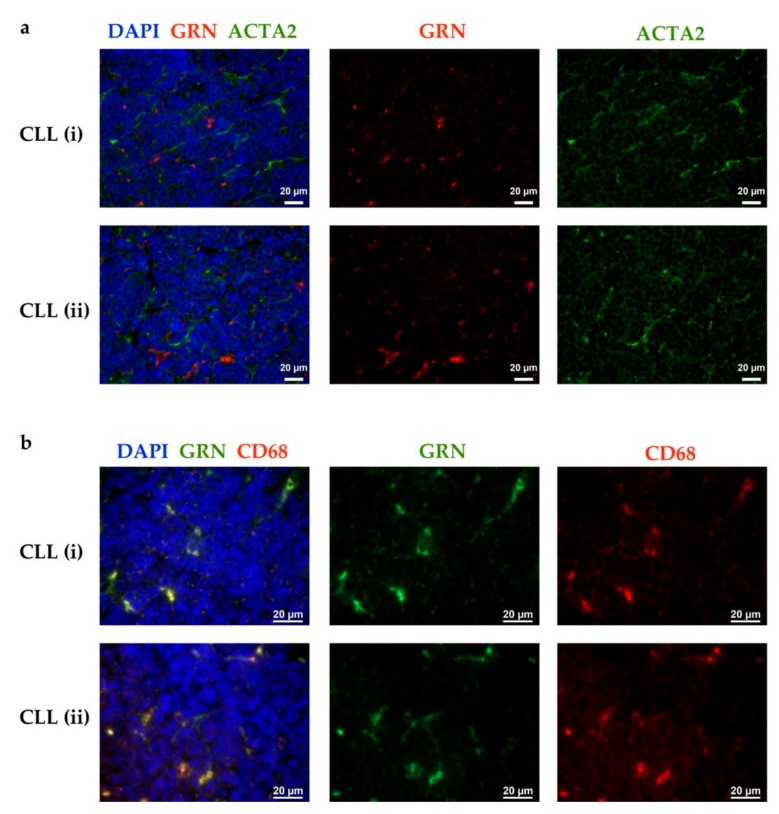
In CLL lymph node (LN) sections, GRN is detected in macrophages. CLL patient-derived LNs were analyzed for GRN and ACTA2 or CD68 by immunofluorescent stainings. Merged (left) and single channel (middle and right) images of 2 representative patients are shown (i and ii). Data display including brightness and contrast was adjusted individually in all images (no quantitative comparability). Nuclei are shown in blue (DAPI). (**a**) GRN (red) and ACTA2 (green) co-staining. Representative for lymph nodes from n = 4 CLL patients. (**b**) GRN (green) and CD68 (red) co-staining. Representative for lymph nodes from n = 6 CLL patients.

To circumvent alloantigen-mediated rejection of transplanted Eμ-*TCL1* cells, the use of recipient mice with cell type-restricted knockout for *Grn* was necessary, to allow exposure of T cells in recipient mice to GRN when undergoing physiological tolerance mechanisms. Therefore, we had to identify the main cellular source of GRN in CLL in vivo. By immunofluorescence stainings of human CLL patient-derived lymph node sections, GRN was detected in irregularly shaped cells exhibiting cellular extensions, which did not resemble lymphocytes (Figure 4). Co-staining with an anti-α-smooth muscle actin antibody, used as activated fibroblast or CAF marker, indicated that the GRN positive cells were not activated fibroblasts (Figure 4a). Instead, GRN staining co-localized with CD68, a marker for macrophages (Figure 4b).

Based on the detection of GRN in CLL lymph nodes and published data proposing that GRN is predominantly expressed by immune cells [90,91], we generated bone marrow chimeric mice that lack GRN in hematopoietic cells. Thus, *Grn*^−/−^ bone marrow cells were transplanted to lethally irradiated syngeneic wild type mice. To generate a control group, the same procedure was performed with bone marrow derived from wild type mice. 10 weeks later and after confirming bone marrow reconstitution, Eµ-*TCL1* adoptive transfer was performed to *Grn*^−/−^ and wild type chimeras and compared to non-transplanted chimeras.

**Figure 5:**
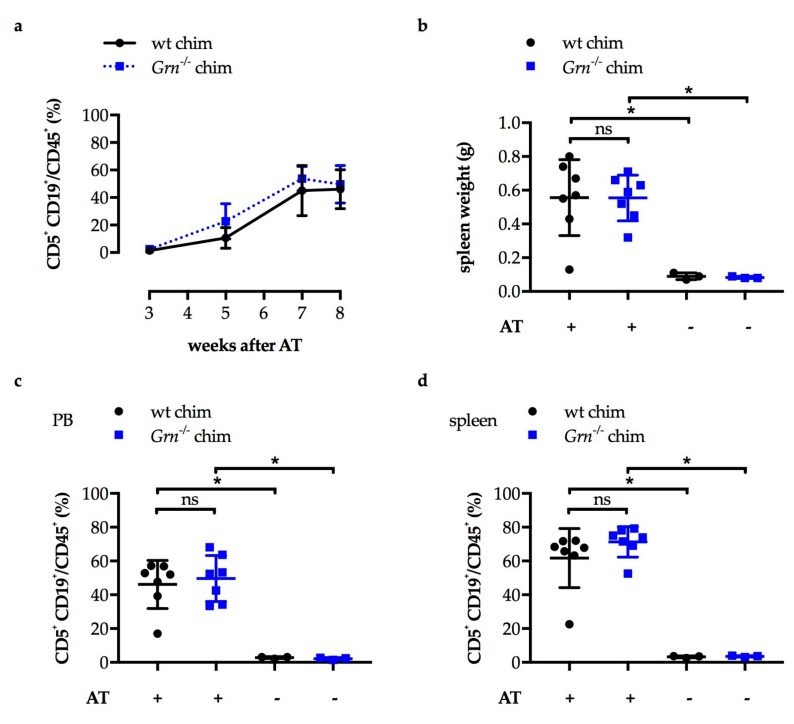
After Eµ-*TCL1* adoptive transfer, leukemia develops equally in *Grn*^−/−^ bone marrow chimeras and controls. 1 × 10^7^ Eµ-*TCL1* tumor cells were adoptively transferred (AT) to n = 7 *Grn*^−/−^ bone marrow chimeras (*Grn*^−/−^ chim) and n = 7 wild type bone marrow chimeras (wt chim) by i.v. injection. n = 3 of each chimeras were left non-transplanted as controls. All chimeric mice were females and 20-weeks old at the time of AT. Means and SD are depicted. (**b**–**d**) were analyzed by Mann-Whitney U tests of indicated pairs, * *p* < 0.05, ns: not significant. (**a**) Leukemic cell load (CD5^+^ CD19^+^ cells out CD45^+^ cells) in the peripheral blood (PB) was analyzed by flow cytometry at different time points after AT. Mann-Whitney U tests comparing both experimental groups at each time point, all *p* values > 0.05. (**b**) Spleen weights were assessed at the study endpoint 8 weeks after AT. (**c**) Leukemic cell load (CD5^+^ CD19^+^ cells out CD45^+^ cells) in the PB was analyzed by flow cytometry at the study endpoint. (**d**) Leukemic cell load (CD5^+^ CD19^+^ cells out CD45^+^ cells) in splenic single cell suspensions was analyzed by flow cytometry at the study endpoint.

Unlike in the first study, leukemic cell load in the blood increased equally over time in both *Grn*^−/−^ chimeras and wild type chimeras (Figure 5a). At the study endpoint, 8 weeks after Eµ-*TCL1* adoptive transfer, mean spleen weight (Figure 5b), leukemic cell load in the blood (Figure 5c) and in the spleen (Figure 5d) were increased in the adoptively transplanted mice compared to the respective non-transplanted controls. There was no significant difference in spleen weight, and in leukemic cell load in blood and spleen detected between *Grn*^−/−^ chimeras and wild type chimeras (Figure 5b–d). In conclusion, knockout of GRN in hematopoietic cells did not impact on CLL development in vivo.

## 3. Discussion

In this study, we identify GRN to be highly elevated in CLL based on its quantification in the serum of 249 patients with an advanced stage of disease (Binet B and C). Our results thereby complement and extend an independent study by Göbel et al. including mainly CLL patients with low clinical stage (Binet A) [65]. We demonstrate that high GRN is associated with an increased risk of CLL progression and patients´ death and correlates with established negative prognostic markers. Along this line, it was earlier observed in consecutive measurements of individual CLL patients that increasing GRN levels were dynamically accompanied by rising blood lymphocyte counts [65]. Collectively, the studies suggest the determination of serum GRN, as an easily assessable marker, for patient-individual prognostications. It remains to be tested how GRN integrates into prognostic indices containing multiple variables that are currently arising in clinical practice, such as the CLL-IPI [92].

GRN was previously reported to be expressed by CLL cells and upregulated in prognostically less favorable ZAP70^+^ CD38^+^ compared to ZAP70^−^ CD38^−^ CLL cells in cDNA microarray studies performed with CLL cell preparations that exhibited >93% CD19^+^ cells [65,93]. Surprisingly, we detected GRN predominantly in macrophages in CLL lymph node sections. Published data support our finding of myeloid cells as a source of GRN. For example, microglia which are resident macrophages of the brain, strongly express GRN and upregulate it upon their activation [94,95]. Interestingly, deregulated microglial activation plays a key role in neurodegenerative diseases caused by GRN deficiency [73,96]. In pancreatic cancer, Nielsen et al. demonstrated, in line with our data, that GRN is highly expressed by liver metastasis-associated macrophages [81]. In vitro experiments further revealed that macrophages upregulated GRN expression when they were cultured in pancreatic cancer cell line-conditioned medium or stimulated to acquire an M2-like phenotype [81]. As CLL was shown to be associated with an accumulation of monocytes and a tumor-protective myeloid cell skewing, this suggests that GRN secretion by tumor-educated myeloid cells confounds GRN elevation in CLL patients’ serum [15,21,24].

There were several reasons why GRN appeared to be an ideal candidate to be tested as a driver of CLL and potential novel therapeutic target. First, its association with increased risk of progression and death in CLL suggested a tumor-promoting role. Second, published data had shown its upregulation also in other cancers, and proposed immunoregulatory and growth promoting functions. However, in contrast to the hypothesis, in our experiments presented in this manuscript GRN did neither support CLL cell survival or proliferation in vitro nor did *Grn* knockout in the hematopoietic compartment affect CLL development in vivo.

How can we explain that GRN exhibits tumor-promoting functions in other cancers [68,69,70] but does not impact on neoplastic capabilities of CLL cells? First, upstream receptors that mediate oncogenic properties and signaling differ between tumors. GRN was, for example, suggested to induce PI3K/AKT pathway activation by binding to the receptor tyrosine kinase EPH receptor A2 (EPHA2), which was linked to promoting the growth of diverse solid tumors [67]. In contrast, activation of the PI3K/AKT pathway in CLL is induced by other well-known stimuli, particularly through B cell receptor signaling [97], while EPHA2 expression in hematopoietic cells is low [91] and its functional role thus questionable. Second, even if proposed GRN binding partners are known to be relevant in CLL, culture conditions that are used for their testing differ between studies. For example, GRN was found to co-activate toll-like receptor 9 (TLR9), which is highly expressed by CLL cells and can convey proliferative signals [74,83,84]. However, in contrast to our set-up, this capability of GRN was demonstrated in murine macrophages, and only detectable when GRN was cleaved by endogenous proteases, which may not be present in human CLL cell cultures [74]. Co-stimulation of CLL cells with the TLR9 agonist CpG and GRN in which we did not observe an additive effect on cell proliferation suggested, that this interaction does not play a significant role in human CLL cells in vitro. However, whether in vivo additional factors, such as proteases that cleave GRN, have an influence is hard to predict.

In addition, in vivo experiments utilizing the Eµ-*TCL1* adoptive transfer CLL mouse model reinforced that GRN deficiency in the hematopoietic compartment does not significantly impact on CLL development. A systemic GRN knockout in recipient mice most likely caused a rejection of Eµ-*TCL1* cells due to their GRN expression as we have recently discussed [89], and was thus not suitable to draw reliable conclusions on the function of GRN. It has to be considered, that in a knockout model restricted to specific cell-types, GRN-proficient cells might be able to compensate for the loss, as for example seen in a microglia-specific GRN knockout model that did not recapitulate the neurodegenerative phenotype of systemic GRN knockout mice, likely due to neuronal GRN expression [98]. On the other hand, in a study more analogous to ours, Nielsen et al. demonstrated that the sizes of metastatic liver and lung lesions in a mouse model of pancreatic cancer based on tumor cell transplantation were significantly reduced in *Grn*^−/−^ bone marrow chimeric mice compared to controls [81]. Thus, *Grn*^−/−^ bone marrow chimeric mice are indeed a suitable model to detect a potential effect of GRN on cancer growth.

As GRN was associated with regulating the lysosomal pathway in studies on neurodegenerative diseases [55,73,96,99], increased cytoplasmic GRN levels could be of relevance in CLL and one can hypothesize, that secreted GRN is just a side effect. In fact, lysosomal functions are more and more recognized to be particularly crucial for cancer cells influencing many cellular functions critical for malignant cells, such as proliferation, differentiation, and autophagy [100]. To test the hypothesis of whether CLL is driven by intracellular GRN within CLL cells, GRN-deficiency of tumor cells needs to be achieved, for example by crossing transgenic Eμ-*TCL1* with *Grn*^−/−^ mice, or by disrupting *GRN* in human CLL cells in vitro. However, first, our data showing that GRN in CLL is largely accessory-cell derived argues against a primary function of cytoplasmic GRN within CLL cells. Second, and most relevantly, independent of a possible intracellular function, our data indicate that there is no rationale to antagonize the elevated extracellular serum GRN as a new approach in CLL therapy.

Although GRN was previously shown to induce CAF-like phenotypes in human mammary fibroblasts [57] and murine hepatic stellate cells [81], the induction of a CAF-like gene expression pattern in human MSCs in CLL co-culture was not recapitulated by GRN exposure of MSCs. Given the variety in originating cells and the growing evidence of heterogeneity in CAF phenotype and function [101,102,103], it is not surprising that several mechanisms for their formation exist. For CLL, it was for example recently demonstrated that extracellular vesicles derived from the CLL cell line MEC1 can induce CAF differentiation of MSCs [46]. However, only 37 of the here presented genes assigned to 454 Illumina probes that are significantly deregulated in MSCs by CLL co-culture are also found in the published transcriptional signature of the vesicle-induced CAFs. This indicates, that (in addition to potential differences between extracellular vesicles from MEC1 and primary CLL cells) CLL-mediated CAF induction from MSCs is not solely mediated by vesicles. Also, CLL-derived soluble factors, such as platelet-derived growth factor (PDGF) [104], and cell contact-dependent mechanisms [105] were described to impact on stromal cell phenotypes. Collectively, CLL-specific CAF induction from MSCs is likely mediated by a combination of signals, which may be even more pronounced in vivo, and GRN does not appear to play a significant role in this.

In addition to questioning the function of GRN in CLL, our study provides a comprehensive analysis of CLL-specific CAFs generated from MSCs. In line with our transcriptome data, factors including CXCL8, CXCL1, MMP3, and MMP1 were previously detected in CLL-stroma co-culture supernatants by cytokine arrays [20]. The upregulation of inflammatory cytokines and extracellular matrix-degrading proteases complies with features commonly associated with CAFs, which were linked to cancer cell invasion and tumor-promoting immune modulation [33,34,35,36,37,38]. Interestingly, CXCL8 was shown to support the survival of CLL cells cultured in vitro [106,107], and reported to be upregulated in the plasma of CLL patients [108]. It therefore not only comprises immune-regulatory functions, but also a direct pro-tumorigenic role of CLL-specific CAFs. Also, proteases can harbor pleiotropic functions. For example, MMP9 was shown to both modulate local microenvironmental niches in CLL [109] and exhibit direct CLL cell survival promoting properties [110,111]. It remains to be tested, whether the proteases identified in our data have a similar effect on CLL cell viability.

In the future, it has to be clarified which role CAFs play for CLL in vivo. We and others have shown that ACTA2 positive cells, identified as CAFs in other cancers, are found in CLL lymph nodes [46,112]. A more detailed analysis of CAFs in murine and human CLL specimens, including a broad set of phenotypical markers, like recently done in breast cancer [102], will help to better characterize CAF populations in vivo. Here, our CLL-specific CAF gene expression profile can serve as a reference. To test the role of MSCs in particular, their recruitment from the bone marrow can be investigated using bone marrow chimeric mice with labeled bone marrow cells as recipients for Eμ-*TCL1* adoptive transfer, similarly to previous studies in models of gastric [39] and breast cancer [40]. In addition, co-injection experiments of manipulated or pretreated MSCs with CLL cells to mice have the potential to elucidate their function in vivo, analogous to previous studies on other cancers [41,42,113]. In conclusion, even though not suggesting a functional role for the elevated GRN serum levels in CLL, the data provided in this report characterize CLL specific CAFs and represent a basis for future studies investigating their pathological relevance.

## 4. Materials and Methods

### 4.1. Human Samples

Peripheral blood and serum was obtained from CLL patients at Ulm University Hospital, at Heidelberg University Hospital, or enrolled in the CLL8 study by the German CLL Study Group (ClinicalTrials.gov: NCT00281918). All CLL patients matched the diagnostic criteria of the World Health Organization. CLL patient characteristics are summarized in Supplementary Tables S1 and S3. CLL patient-derived paraffin embedded lymph node sections were kindly provided by Prof. Dr. Andreas Rosenwald, Institute of Pathology, University of Würzburg, Würzburg, Germany. Bone marrow aspirates that served for isolation of MSCs were obtained from healthy donors at Heidelberg University Hospital. All samples were obtained after written informed consent was provided by the donors and approval was given by the local ethics committees or institutional review boards in Heidelberg (ethical approval codes 52/98, S-254/2016, and S-348/2004), Ulm (ethical approval codes 93/12 and 96/08), Würzburg (ethical approval from 01/2006), or of each institution participating in the CLL8 study.

### 4.2. Quantification of Progranulin in Serum Samples

GRN was quantified in serum samples from 249 CLL patients from the CLL8 study cohort and from 42 age- and sex-matched healthy donors by cytometric bead arrays using the human soluble protein master buffer kit (BD Biosciences, Heidelberg, Germany) according to the manufacturer’s instructions. Serum samples were analyzed in 1:2 dilution. Capture beads were synthesized by coupling anti-GRN capture antibodies (human Progranulin Duoset, R&D Systems, Minneapolis, MN, USA) to functionalized beads (BD Biosciences). A biotinylated detection antibody (Duoset, R&D Systems) and a streptavidin-PE conjugate (BD Biosciences) were used for visualization. Data was acquired on a FACS Canto II flow cytometer and analyzed with FCAP software (BD Biosciences).

### 4.3. Isolation of Human CLL Cells

Peripheral blood mononuclear cells (PBMCs) were isolated from CLL patient-derived EDTA-anticoagulated blood samples by density gradient centrifugation using Biocoll separating solution (Biochrom/Merck, Berlin, Germany) and Leucosep centrifuge tubes (Greiner Bio-One, Kremsmünster, Austria). CLL cell content of PBMCs defined as CD5^+^ CD19^+^ cells out of single cells was determined by flow cytometry and is listed in Supplementary Table S3. If indicated, PBMCs were further enriched for CLL cells by magnetic-activated cell sorting (MACS) using CD19 microbeads, MACS LS columns, MACS buffer, and a QuadroMACS separator (all Miltenyi Biotec, Bergisch Gladbach, Germany) according to the manufacturer´s instructions. Thereby, purities of >98% were reached as determined by flow cytometry (CD5^+^ CD20^+^ cells out of single cells).

### 4.4. Isolation of Human MSCs

MSC isolation was performed as previously described [114,115]. In brief, mononuclear cells were purified from healthy donor-derived bone marrow aspirates by density gradient centrifugation and seeded in a specific growth medium (MSCGM Bulletkit, Lonza, Walkersville, MD, USA) at 10^5^ cells/cm^2^. The presence of mesenchymal (CD73, CD90, CD105, CD44) and absence of hematopoietic surface markers (CD45, CD11b, CD19, HLADR) were confirmed by flow cytometry prior to usage in experiments.

### 4.5. Flow Cytometry

A List of antibodies and a summary of staining panels is provided in the supplements (Supplementary Tables S5 and S6). Unstained samples, single stainings, isotype, and FMO (fluorescence minus one) controls were set up to enable proper gating, compensation, and normalization depending on the antibody panels. All samples were analyzed at a BD LSR Fortessa or BD FACS Canto II flow cytometer (BD Biosciences), data were processed using BD FACS Diva (BD Biosciences) and FlowJo V10 software (FlowJo, Ashland, OR, USA).

Human single-cell suspensions were Fc blocked by IgG from human serum (Sigma-Aldrich/Merck, Darmstadt, Germany), followed by incubation with fluorochrome-coupled antibodies in indicated dilutions for 15 min at 4 °C in the dark. FACS buffer for human cells consisted of PBS, 2% fetal calf serum (FCS), and 0.02% sodium azide. As an exception, human MSCs were not FC blocked. MSCs were detached using accutase solution (Sigma-Aldrich/Merck) prior to stainings.

Murine single-cell suspensions were labeled by incubation with fluorochrome-coupled antibodies in indicated dilutions for 30 min at 4 °C in the dark. FACS buffer for murine cells consisted of PBS with 5% FCS, or pure PBS if a fixable viability dye was used. If cells were not analyzed immediately, they were fixed using IC fixation buffer (eBioscience/Thermo Fisher Scientific, Waltham, MA, USA). Leukemic cell load in the blood was determined by whole blood staining. EDTA-anticoagulated blood was diluted 1:2 or 1:4 with PBS and incubated with fluorochrome-coupled antibodies in indicated dilutions for 30 min at 4 °C in the dark. Red blood cells were then lysed using 1-step lyse and fix solution (eBioscience/Thermo Fisher Scientific) or BD FACS lysing solution (BD Biosciences).

### 4.6. Functional In Vitro Studies with CLL Cells

CLL cells were cultured in DMEM supplemented with 4.5 g/L glucose, 4 mM l-glutamine, 10% FCS, and 1% Penicillin/Streptomycin (10,000 U/mL), with or without recombinant human GRN (R&D Systems, Minneapolis, MN, USA) addition.

#### 4.6.1. Annexin V/7AAD Apoptosis Assay

CLL cells were cultured under different conditions: (i) 1 × 10^6^ CLL PBMCs/mL (low density) were cultured for 48 h, with or without 1 µg/mL GRN. (ii) 2.5 × 10^7^ CLL PBMCs/mL (high-density) were cultured for 9 days, with or without 1 µg/mL GRN added daily. (iii) 4 × 10^6^ CLL PBMCs/mL were cultured with 50 U/mL IL4 (Miltenyi Biotec) for 48 h, with or without 1 µg/mL GRN. (iv) 1 × 10^6^ CLL PBMCs/mL were cultured with 2 µM CpG oligodeoxynucleotide (DSP30, sequence 5′ to 3′ TCGTCGCTGTCTCCGCTTCTTCTTGCC) and 100 U/mL IL2 (Miltenyi Biotec, Bergisch Gladbach, Germany) for 4 days, with or without 5 µg/mL GRN.

Viability of CLL cells was assessed by Annexin V/7AAD apoptosis assay by flow cytometry as previously described [19], using Annexin V PE, 7-Aminoactinomycin D (7AAD), and Annexin V binding buffer (all from BD Biosciences). Annexin V^−^ 7AAD^−^ cells were defined as viable cells.

#### 4.6.2. CellTiter-Glo Luminescent Viability Assay

3 × 10^5^ CLL PBMCs in 100 µL were seeded in 96-well luminescence culture plates (white color, Thermo Fisher Scientific, Waltham, MA, USA). In the experimental groups, media were supplemented with 1 or 4 µg/mL recombinant GRN. The CellTiter-Glo luminescent cell viability assays (Promega, Madison, WI, USA) were performed after 48 h according to the manufacturer’s protocol and analyzed using a Mithras LB 940 microplate reader (Berthold Technologies, Bad Wildbad, Germany).

#### 4.6.3. Click-iT EdU Proliferation Assay

5 × 10^5^ freshly isolated CLL PBMCs were cultured in 500 µL FCS-free growth medium and stimulated with 2 µM CpG oligodeoxynucleotide (DSP30, sequence 5′ to 3′ TCGTCGCTGTCTCCGCTTCTTCTTGCC) and 100 U/mL IL2 (Miltenyi Biotec), as previously described [83,84], for 4 days. Additionally, 200 ng/mL or 1 µg/mL GRN were administered at day 0, 1, 2, and 3. The proliferation of CLL cells was determined by a Click-iT EdU assay protocol according to modified instructions of the manufacturer using the Click-iT EdU alexa fluor 488 flow cytometry assay kit and Click-iT cell reaction buffer kit in combination with the alexa fluor 488 azide dye (all from Invitrogen/Thermo Fisher Scientific, Waltham, MA, USA). 24 hours before harvesting, the cells were incubated in 10 µM of the nucleoside analog EdU to label the proliferating fraction. In brief, at day 4, cells were harvested, fixed in 4% paraformaldehyde, permeabilized by incubation in PBS containing 0.1% Triton X-100, and the incorporated EdU was detected using the alexa fluor 488 azide dye in combination with components of the Click-iT cell reaction buffer kit. Samples were analyzed by flow cytometry. Alexa fluor 488^+^ cells were defined as proliferating cells.

#### 4.6.4. CLL Cell Activation Assay

4 × 10^6^ CLL PBMCs/mL were stimulated with 100 ng/mL CD40 mega-ligand (CD40ML, Enzo Life Sciences, Lörrach, Germany) and 50 U/mL IL4 (Miltenyi Biotec). Cells were harvested after 48 h to analyze CD86 surface expression as activation marker by flow cytometry.

### 4.7. Human MSC Cultures and Their Characterization

#### 4.7.1. MSC Cultures

MSCs were cultured in MSC-growth medium (MSCGM Bulletkit, Lonza, Walkersville, MD, USA) in the following conditions. (i) Samples for gene expression profiling by Illumina BeadChip arrays (Figure 3a,b): 5 × 10^5^ MSCs were cultured in 2 mL MSCGM in 6 cm dishes for 5 days. MSCs were either left untreated, treated with 3 µg/mL GRN (R&D Systems) daily or co-cultured with 5 × 10^6^ CD19 MACS-purified CLL cells. (ii) Samples for gene expression analysis by quantitative real-time PCR (qPCR): 5 × 10^5^ MSCs were cultured in 1 mL MSCGM in 6-well plates or in 2 mL MSCGM in 6 cm dishes for 3 days. MSCs were either left untreated, treated with 3 µg/mL GRN daily, or co-cultured with 1 × 10^6^ CD19 MACS-purified CLL cells.

#### 4.7.2. RNA Isolation

CLL cells were thoroughly removed from the co-cultures by repetitive washing with PBS. To obtain RNA, adherent MSCs were lysed with 1 ml TRIzol reagent (Invitrogen/Thermo Fisher Scientific) directly in the culture dishes and purified by adding 300 µL chloroform and centrifugation according to manufacturer’s protocol. RNA was subsequently further purified using the Qiagen 74004 RNeasy Micro Kit (Quiagen, Hilden, Germany). RNA concentrations and quality were determined by a NanoDrop ND-1000 spectrometer (Thermo Fisher Scientific).

#### 4.7.3. Quantitative Real-Time PCR (qPCR)

RNA was purified by DNA digestion and transcribed to cDNA by reverse transcription making use of the DNase I kit and SuperScript II reverse transcriptase kit, respectively (both Invitrogen/Thermo Fisher Scientific). Quantitative real-time polymerase chain reaction (qPCR) was performed using the Absolute SYBR Green ROX Mix (Thermo Scientific/Thermo Fisher Scientific, Waltham, MA, USA) as DNA intercalating dye and the ABI Prism 7900HT sequence detection system (Applied Biosystems/Thermo Fisher Scientific, Waltham, MA, USA) as previously described [19]. Exon spanning primers for genes of interest and multiple reference genes were used (Supplementary Table S7), which were partly derived from databases (RTPrimerDB, PrimerBank) [116,117]. For each primer pair a dilution series of cDNA from Stratagene human reference RNA (Agilent Technologies, Santa Clara, CA, USA) was included to enable efficiency check. Nuclease-free water (Ambion/Thermo Fisher Scientific, Waltham, MA, USA) and 15 ng genomic DNA (human placenta, BioChain, Hayward, CA, USA) were applied as negative controls. Gene expression analysis was performed according to the ΔΔCT method [118], resulting fold changes were log_2_ transformed.

#### 4.7.4. Genome-Wide Gene Expression Direct Hybridization

Gene expression profiling was kindly performed by the Genomics and Proteomics Core Facility of the German Cancer Research Center (DKFZ), Heidelberg, Germany, using an Illumina HumanHT-12 v4 Expression BeadChip (Illumina, San Diego, CA, USA) after submission of 12 DNase-treated RNA samples. All samples were included in one chip.

#### 4.7.5. Protein Quantifications in the Cell Culture Supernatants

MMP1 was quantified by the human MMP1 enzyme-linked immunosorbent assay (ELISA) kit (Abcam, Cambridge, United Kingdom) according to manufacturer’s instructions in undiluted samples. Absorbance was analyzed by a Mithras LB 940 microplate reader (Berthold Technologies). CXCL8 was quantified by cytometric bead array (CBA) using the human IL-8 flex-set and BD CBA human soluble protein master buffer kit (BD Biosciences) following the manufacturer’s protocol. Here, cell culture supernatants were applied in 1:50 dilution to range within the standard curve. Data was acquired on a FACS Canto II flow cytometer and analyzed with FCAP software (BD Biosciences). Protein concentrations produced by 2.5 × 10^5^ MSCs/ml were calculated.

### 4.8. Immunofluorescent Stainings of Human Lymph Nodes

Paraffin-embedded lymph node sections were deparaffinized, incubated in citrate buffer (10 mM sodium citrate) for antigen retrieval, and permeabilized in TTBS (10 mM Tris, 150 mM NaCl, 0,05% Tween20). 0.1% Sudan black B (Sigma-Aldrich/Merck, Darmstadt, Germany) in 70% ethanol was applied as described before to reduce autofluorescence [119]. After washing with TTBS, sections were blocked with 5% normal horse serum (Invitrogen/Thermo Fisher Scientific, Waltham, MA, USA) in TTBS. Primary antibodies listed in Supplementary Table S8 were diluted in TTBS, administered to the slides, and incubated overnight at 4 °C. The next day, slides were washed with TTBS and secondary antibodies were added to incubate for 45 min at room temperature in the dark. Slides were washed and mounted using mounting medium with DAPI (SouthernBiotech, Birmingham, AL, USA) to stain nuclei. Images were acquired using a Zeiss cell observer widefield microscope and ZEN acquisition software (both Carl Zeiss, Oberkochen, Germany).

### 4.9. Mouse Models

Eµ-*TCL1* mice on a C57BL/6 background [87], *Grn*^−/−^ mice on a C57BL/6 background [120], and C57BL/6 wild type mice were used in this study. All mouse experiments were conducted according to governmental and institutional guidelines and authorized by the local authorities (Regierungspräsidium Karlsruhe, permit numbers: G-36/14, G-16/15, G-25/16).

To generate bone marrow chimeric mice, bone marrow cells were isolated from 11-weeks old, female *Grn*^−/−^ mice (n = 2) and 10-weeks old, female wild type mice (n = 2). Bone marrow cells were T cell-depleted by MACS using CD90.2 microbeads (Miltenyi Biotec) according to the manufacturer’s protocol. Purity staining by flow cytometry revealed 98.2% and 98.6% CD3^−^ CD45^+^ cells out of total single cells from *Grn*^−/−^ mice and wild type mice, respectively. Bone marrow cells were transplanted to 10-weeks old, female C57BL/6 wild type acceptor mice (n = 20) by i.v. injection using 5 × 10^6^ cells/mouse 48 h after their gamma-irradiation with two doses of 500 cGy (3 h apart). Bone marrow reconstitution was confirmed after 9 weeks by analysis of the leukocyte composition in the blood by flow cytometry.

Eµ-*TCL1* adoptive transfer was performed to *Grn*^−/−^ bone marrow chimeras and wild type chimeric controls 10 weeks after both were generated, n = 7 per group. Therefore, Eµ-*TCL1* splenocytes derived from a leukemic mouse were enriched for B cells by pan-B cell isolation kit (Stemcell Technologies, Vancouver, BC, Canada) according to manufacturer’s instructions to eliminate non-malignant, non-GRN-deficient cells. This typically resulted in purities (CD5^+^ CD19^+^ cells out of viable CD45^+^ cells) of >95%. Finally, 1 × 10^7^ B cell-enriched splenocytes/mouse were adoptively transferred by i.v. injection.

Peripheral blood of Eµ-*TCL1* adoptively transferred mice was drawn by submandibular vein-puncture and collected in EDTA-coated tubes (Sarstedt, Nümbrecht, Germany). At the study endpoints, blood was collected by heart puncture after mice were sacrificed. Spleens were collected from sacrificed mice, weighted, and processed to single cell suspensions using a gentleMACS dissociator and gentleMACS C-tubes (both from Miltenyi Biotec). Subsequently, red blood cell lysis was performed in ACK lysis buffer (150 mM NH_4_Cl, 10 mM KHCO_3_, 0.1 mM EDTA) for 5–8 min. Cells were then washed with PBS and filtered by using 70 µm cell strainers (BD Biosciences).

### 4.10. Statistical Analysis

To analyze the mutual relation between GRN and β2-microglobulin or thymidine kinase, log_2_ transformed serum concentrations were investigated using Pearson’s correlation. The influence of GRN levels on patient prognosis (with endpoint progression-free survival (PFS), overall survival (OS), or tumor-associated deaths (TAD)) was analyzed by univariate and multivariate Cox proportional hazards regression models. TAD was analyzed with competing risk models. The multivariate analysis included the co-variables rituximab treatment, age, 11q deletion, 17p deletion, and IGHV mutational status. GRN levels were again log_2_ transformed and entered as continuous variables. Due to data unavailability for these co-variables for n = 21 out of n = 249 patients, the multivariate analysis was performed after multiple imputation of missing values by Multivariate Imputations by Chained Equations (MICE) with m = 10 imputed data sets. The association of PFS, OS or TAD with log_2_ GRN levels as a continuous predictor was plotted by means of Stone-Beran plots using symmetrical nearest neighborhoods around the lowest, the median, and the highest observed values [121,122,123]. Statistical analyses of the effects of GRN on survival endpoints were performed with the R statistical software, version 3.5.3 [124], and add-on packages survival version 2.43-3, and mice version 3.3.0 for the multiple imputation of missing values.

Analysis of gene expression data was performed based on unique Illumina probes (instead of transcripts or genes). First, samples were processed by background correction and detection filtering to exclude non-expressed probes. Pairwise comparison of untreated (MSCs cultured alone) versus treated samples (MSCs in CLL co-culture or GRN treated MSCs) was then performed using the empirical Bayes approach by Smyth [125]. For each probe a linear model was fitted with factors characterizing treatment and donor. Significant transcripts were selected based on the moderated t statistic. The Benjamini-Hochberg correction was applied in order to control the false discovery rate.

Other experimental data were analyzed using Prism 7 software (GraphPad Software, San Diego, CA, USA). Data that did not follow a Gaussian distribution, or was scaled as percentage or concentration was analyzed by nonparametric tests. Two groups were either compared by Mann-Whitney U test (unpaired data) or Wilcoxon matched-pairs signed rank test (paired data) depending on experimental design. Log_2_ transformed gene expression changes obtained by qPCR (ΔΔCT method [118]) were statistically analyzed by one-sample t tests comparing the mean of biological replicates to 0 (=no gene expression change relative to untreated control). All statistical tests were performed as two-sided tests. Choosing a confidence level of 95%, *p* values < 0.05 were considered statistically significant and encoded as follows: * *p* < 0.05, ** *p* < 0.01, *** *p* < 0.001, **** *p* < 0.0001.

## 5. Conclusions

Serum GRN is highly elevated and associated with an increased hazard for disease progression and death in CLL. After systematically analyzing the effect of GRN on malignant and microenvironmental cells in vitro, and investigating the consequences of GRN deficiency on CLL-like disease development in the Eµ-*TCL1* adoptive transfer mouse model in vivo, we conclude, that there is no evidence that elevated GRN is a functional driver of CLL.

## Supplementary Materials

The following information is available online at https://www.mdpi.com/2072-6694/11/6/822/s1, Figure S1: GRN secretion by stromal cells of mesenchymal origin is induced in CLL co-cultures, Figure S2: *Grn*^−/−^ mice do not develop leukemia after Eµ-*TCL1* adoptive transfer, Table S1. Characteristics of 249 CLL patients from the German CLL8 study cohort, Table S2: Prognostic value of GRN serum levels in CLL patients, Table S3: CLL patient data (in vitro experiments), Table S4: Top upregulated genes in human mesenchymal stromal cells (MSCs) after CLL co-culture, Table S5: List of flow cytometry antibodies, Table S6: List of flow cytometry antibody panels, Table S7: List of qPCR primers, Table S8: List of antibodies used for immunofluorescent stainings of human CLL lymph nodes. Supplementary Methods.
