# Supplementary material for: Dissecting the Prognostic Significance and Functional Role of Progranulin in Chronic Lymphocytic Leukemia
Source: Cancers (Basel). 2019 Jun 13;11(6):822. doi: 10.3390/cancers11060822 (PMC6627891; doi:10.3390/cancers11060822)
Supplement: Supplementary file 1 [file cancers-11-00822-s001.pdf]

Supplementary Material

Dissecting the Prognostic Significance and Functional Role of Progranulin in Chronic Lymphocytic Leukemia

Lena Schulze-Edinghausen, Claudia Dürr, Selcen Öztürk, Manuela Zucknick, Axel Benner, Verena Kalter, Sibylle Ohl, Viola Close, Patrick Wuchter, Stephan Stilgenbauer, Peter Lichter and Martina Seiffert

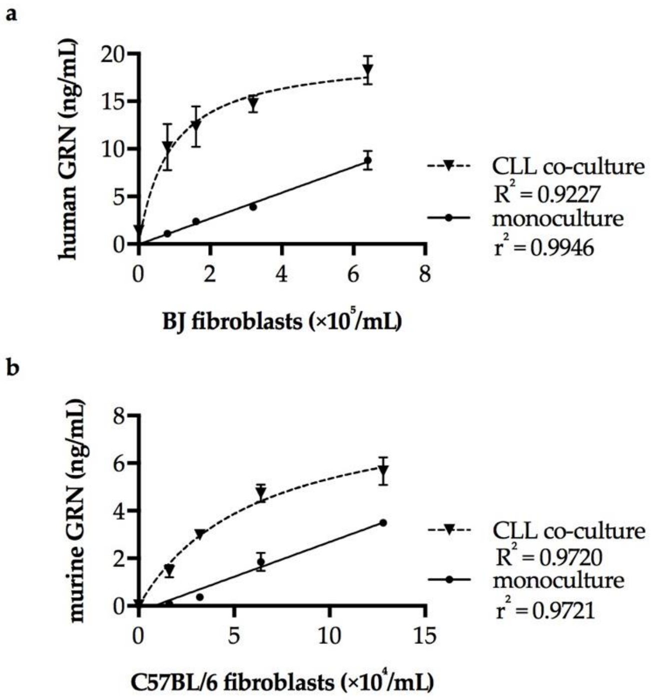

Figure S1. GRN secretion by stromal cells of mesenchymal origin is induced in CLL co-cultures. Increasing numbers of stromal cells were either cultured alone (solid lines) or co-cultured with 2 × 10^6/mL primary human CLL cells (dashed lines) for 5 days. GRN was quantified in cell culture supernatants by ELISA. Means and SD of technical triplicates are plotted. Calculations of linear regression (r^2) and nonlinear regression for a hyperbolic curve (R^2) are depicted. (**a**) Cultures of human BJ fibroblasts. Representative for n = 3 biological replicates of the co-culture. (**b**) Xenogeneic cultures of murine C57BL/6 fibroblasts with and without human CLL cells. GRN was quantified with a murine-specific ELISA kit, the lack of cross-reactivity to human GRN was experimentally validated (not shown). Representative for n = 4 biological replicates of the co-culture.

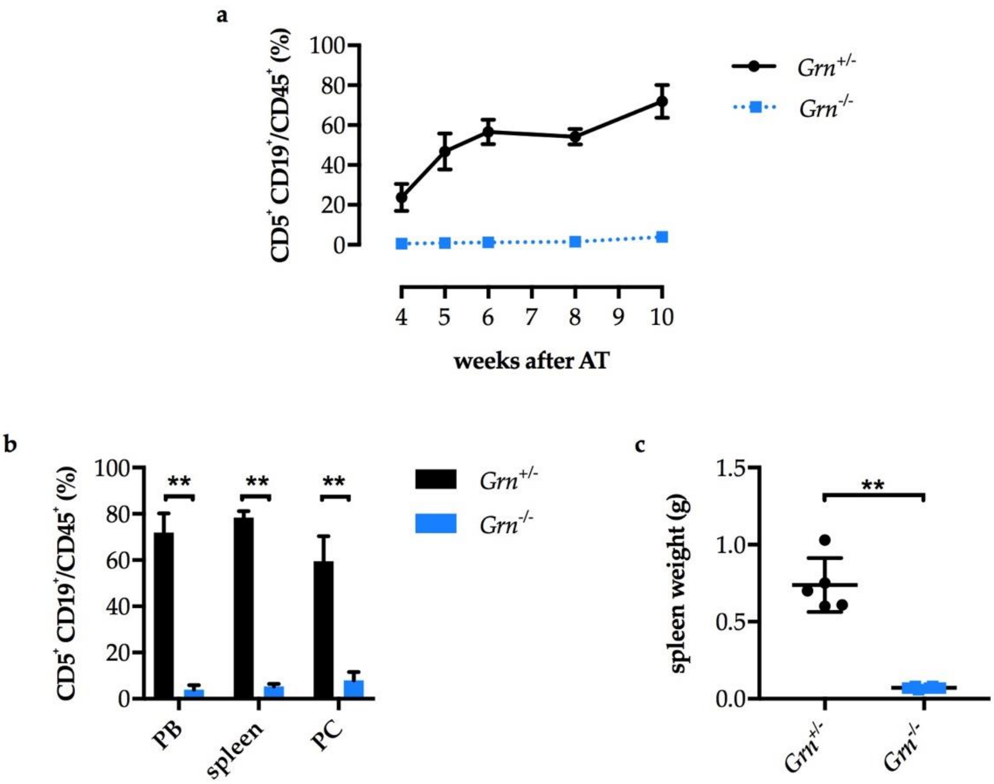

Figure S2. *Grn*$^{-/-}$ mice do not develop leukemia after Eμ-*TCL1* adoptive transfer. 2 × 10^7 Eμ-*TCL1* tumor cells were adoptively transferred (AT) to *Grn*$^{-/-}$ mice (blue) and heterozygous littermates (black) by i.p. injection, all mice were 7–8 week old males, n=5-6 per group. Plots show means and SD. (**a**) Leukemic cell load ($CD5^+$ $CD19^+$ cells out $CD45^+$ cells) in the peripheral blood (PB) was analyzed by flow cytometry at different time points after AT. Mann-Whitney U tests comparing both experimental groups at each time point: $p = 0.0022$ (at 4, 5 and 6 weeks), $p = 0.0043$ (at 8 and 10 weeks). (**b**) Leukemic cell load was analyzed in the PB, in splenic and peritoneal cavity (PC) single cell suspensions at the study endpoint 10 weeks after AT. Mann-Whitney U tests, all $p = 0.0043$. (**c**) Spleen weight was assessed at the study endpoint. Mann-Whitney U test, $p = 0.0022$.

Table S1. Characteristics of 249 CLL patients from the CLL8 study cohort by the German CLL Study Group.

**Patient Characteristics**		**n**	**%**
IGHV status	mutated	89	35.7
	unmutated	149	59.8
	n.a.	11	4.4
TP53 mutation	no	209	83.9
	yes	29	11.7
	n.a.	11	4.4
NOTCH1 mutation	no	150	60.2
	yes	9	3.6
	n.a.	90	36.1
SF3B1 mutation	no	131	52.6
	yes	28	11.2
	n.a.	90	36.1
11q del	no	170	68.3
	yes	64	25.7
	n.a.	15	6.0
17p del	no	217	87.2
	yes	17	6.8
	n.a.	15	6.0
13q del	no	109	43.8
	yes	124	49.8
	n.a.	16	6.4
6q del	no	42	16.9
	yes	0	0
	n.a.	207	83.1
trisomy 12	no	203	81.5
	yes	30	12.1
	n.a.	16	6.4
genetic hierarchy	13q del (sole)	34	13.7
	normal	24	9.6
	trisomy 12	13	5.2
	11q del	34	13.7
	17p del	9	3.6
	other	1	0.4
	n.a.	134	53.8

Serum samples of these patients were used for GRN quantification. IGHV: immunoglobulin heavy-chain variable region gene segments. n.a.: not available. del: deletion. Genetic hierarchy according to Döhner et al. [1]. Patients included in the study comprised clinical stages Binet B and C and required clinical intervention.

Table S2. Prognostic value of GRN serum levels in CLL patients.

	Univariate Analysis				Multivariate Analysis			
	HR	Lower 95 % CI	Upper 95 % CI	*p* Value	HR	Lower 95 % CI	Upper 95 % CI	*p* Value
PFS	1.140	1.049	1.240	0.002	1.0803	0.9818	1.1887	0.113
TAD	1.149	1.027	1.286	0.016	1.0803	0.9506	1.2278	0.237
OS	1.145	1.027	1.276	0.015	1.0687	0.9443	1.2095	0.292

Association of GRN levels with progression-free survival (PFS), tumor-associated deaths (TAD, competing risks analysis), and overall survival (OS) was analyzed by univariate and multivariate Cox proportional hazards regression models. GRN levels were $\log_2$ transformed and entered as continuous variables. The multivariate analysis included the co-variables rituximab treatment, age, 11q deletion, 17p deletion, and IGHV mutational status. Due to data unavailability for these co-variables for n = 21 out of n = 249 patients, the multivariate analysis was performed after multiple imputation of missing values. HR: hazard ratio estimated from Cox proportional regression model. 95 % CI: 95 % confidence interval of the estimated HR. p value: Wald *p* value.

Table S3. CLL patient data.

CLL Patient Number	IGHV Status	FISH	ZAP70 Status	CLL Cell Content	Refers to Figure:
1	mutated	13q del (85 % bi-del)	n.a.	33.9 %	2
2	mutated	13q del	negative	75.9 %	S1
3	mutated	13q del	negative	97.7 %	S1
4	unmutated	13q del	n.a.	88.3 %	S1
5	n.a.	n.a.	n.a.	83.6 %	2
6	n.a.	n.a.	n.a.	97.2 %	S1, 2
7	n.a.	n.a.	n.a.	92.9 %	2
8	n.a.	n.a.	n.a.	78.1 %	2
9	n.a.	n.a.	n.a.	88.4 %	2
10	unmutated	13q del	n.a.	68.0 %	S1
11	n.a.	n.a.	n.a.	89.4 %	3
12	n.a.	n.a.	n.a.	95.9 %	2
13	n.a.	n.a.	n.a.	95 %	3
14	mutated	13q del	negative	82.5 %	3
15	unmutated	normal	n.a.	96.4 %	3
16	unmutated	13q del	n.a.	94.6 %	3
17	mutated	13q del	n.a.	87.1 %	3
18	n.a.	n.a.	n.a.	89.4 %	3
19	n.a.	n.a.	n.a.	80.1 %	2, 3
20	mutated	13q del	n.a.	82.3 %	2
21	mutated	normal	negative	92.7 %	2
22	n.a.	n.a.	n.a.	85.9 %	2
23	mutated	13q del	negative	67 %	2
24	mutated	13q del	n.a.	79.4 %	2
25	unmutated	13q del	n.a.	93.6 %	2
26	mutated	13q del	n.a.	83.4 %	2
27	n.a.	n.a.	n.a.	56.7 %	2
28	n.a.	n.a.	n.a.	79.2 %	2
29	n.a.	n.a.	n.a.	n.a.	2
30	n.a.	n.a.	n.a.	n.a.	2
31	mutated	normal	n.a.	98.2 %	2
32	n.a.	n.a.	n.a.	94.5 %	2

Characteristics of CLL donors that provided blood samples that were used for in vitro experiments. IGHV: immunoglobulin heavy-chain variable region gene segments. FISH: fluorescence in situ hybridization. del: deletion. trans: translocation. bi-del: bi-deletion. n.a.: not available. CLL cell content: $CD5^+$ $CD19^+$ cells out of single cells in patient-derived peripheral blood mononuclear cells as determined by flow cytometry.

Table S4. Top upregulated genes in human mesenchymal stromal cells (MSCs) after CLL co-culture.

Illumina Probe ID	Gene Symbol	Gene ID	log FC	av. expr.	t	*p* value	adj. *p* value	B
ILMN_2313672	IL1RL1	9173	3.84	6.01	9.00	1.56E-04	3.88E-02	1.68
ILMN_1775501	IL1B	3553	3.82	6.53	7.46	4.17E-04	4.29E-02	0.74
ILMN_2188862	GDF15	9518	3.48	11.85	15.03	9.90E-06	3.57E-02	3.95
ILMN_1726448	MMP1	4312	3.41	6.07	14.46	1.22E-05	3.57E-02	3.80
ILMN_2352097	GPR56	9289	3.36	6.84	12.47	2.73E-05	3.57E-02	3.19
ILMN_1676984	DDIT3	1649	3.23	10.73	9.12	1.46E-04	3.88E-02	1.74
ILMN_1661861	CSF2	1437	3.19	6.02	9.27	1.34E-04	3.88E-02	1.82
ILMN_1729691	SLC16A6	9120	3.13	5.71	12.77	2.41E-05	3.57E-02	3.29
ILMN_1758895	CTSK	1513	3.11	10.82	13.20	2.01E-05	3.57E-02	3.43
ILMN_1734611	BDKRB1	623	3.09	7.52	10.64	6.44E-05	3.57E-02	2.48
ILMN_3235379	LOC100134265	100134265	3.05	8.16	10.27	7.77E-05	3.57E-02	2.31
ILMN_1800225	PPARG	5468	3.05	7.51	12.84	2.33E-05	3.57E-02	3.31
ILMN_1677092	GEM	2669	3.03	7.94	7.21	4.95E-04	4.32E-02	0.57
ILMN_1681983	RSPO3	84870	3.03	6.57	6.88	6.29E-04	4.55E-02	0.33
ILMN_2384122	GPR56	9289	3.00	6.44	9.44	1.21E-04	3.88E-02	1.91
ILMN_1780057	RENBP	5973	2.94	5.86	12.26	3.00E-05	3.57E-02	3.11
ILMN_3298423	TOX2	84969	2.88	7.62	12.46	2.74E-05	3.57E-02	3.19
ILMN_1798210	E2F7	144455	2.87	7.64	10.87	5.73E-05	3.57E-02	2.58
ILMN_2400326	DYRK3	8444	2.87	8.97	7.49	4.07E-04	4.28E-02	0.76
ILMN_1773262	ESM1	11082	2.86	8.64	9.52	1.16E-04	3.88E-02	1.95
ILMN_1715401	MT1G	4495	2.80	9.87	11.75	3.77E-05	3.57E-02	2.93
ILMN_2364384	PPARG	5468	2.74	6.16	11.10	5.12E-05	3.57E-02	2.67
ILMN_1837428	n.a.	n.a.	2.73	8.12	11.31	4.63E-05	3.57E-02	2.76
ILMN_1687213	C8orf13	83648	2.71	6.57	8.07	2.78E-04	4.09E-02	1.13
ILMN_1748840	CALB2	794	2.71	10.12	10.10	8.48E-05	3.69E-02	2.23
ILMN_1671353	IL12A	3592	2.70	7.89	11.71	3.84E-05	3.57E-02	2.91
ILMN_1733579	EVI2A	2123	2.68	8.11	11.53	4.18E-05	3.57E-02	2.84
ILMN_3237376	GRAMD1B	57476	2.65	5.93	10.63	6.47E-05	3.57E-02	2.47
ILMN_1730454	FOLR3	2352	2.65	7.39	8.71	1.86E-04	3.97E-02	1.52
ILMN_2212878	ESM1	11082	2.65	9.17	8.98	1.58E-04	3.88E-02	1.66
ILMN_3248511	FAM167A	83648	2.61	6.89	7.78	3.34E-04	4.11E-02	0.95
ILMN_1744217	HTR7	3363	2.61	6.16	10.46	7.05E-05	3.57E-02	2.40

Illumina Probe ID	Gene Symbol	Gene ID	log FC	av. expr.	t	*p* value	adj. *p* value	B
ILMN_2311537	HMGA1	3159	2.59	10.55	10.50	6.89E-05	3.57E-02	2.42
ILMN_1808508	KITLG	4254	2.57	7.24	9.38	1.25E-04	3.88E-02	1.88
ILMN_3258346	LOC100130009	100130009	2.56	6.12	9.90	9.45E-05	3.72E-02	2.14
ILMN_2285708	C6orf48	50854	2.54	6.25	6.96	5.92E-04	4.53E-02	0.39
ILMN_1687978	PHLDA1	22822	2.50	11.62	8.76	1.81E-04	3.97E-02	1.54
ILMN_1699421	ANXA10	11199	2.49	6.69	9.12	1.46E-04	3.88E-02	1.74
ILMN_2341595	KITLG	4254	2.47	7.88	9.95	9.19E-05	3.70E-02	2.16
ILMN_1697409	TNFRSF14	8764	2.46	9.28	10.61	6.53E-05	3.57E-02	2.46
ILMN_1669046	FOXQ1	94234	2.44	6.51	10.09	8.55E-05	3.69E-02	2.23
ILMN_1727592	FRMD5	84978	2.43	6.14	7.28	4.71E-04	4.32E-02	0.62
ILMN_1783840	FLJ42986	389012	2.43	7.12	6.71	7.14E-04	4.69E-02	0.20
ILMN_1761281	LOC441019	441019	2.43	9.33	10.33	7.55E-05	3.57E-02	2.34
ILMN_3289247	LOC400750	400750	2.42	7.54	7.46	4.16E-04	4.29E-02	0.74
ILMN_1712759	SBSN	374897	2.41	5.88	9.26	1.35E-04	3.88E-02	1.81
ILMN_1800091	RARRES1	5918	2.40	8.10	10.35	7.47E-05	3.57E-02	2.35
ILMN_1656593	AK5	26289	2.39	6.90	7.30	4.64E-04	4.31E-02	0.63
ILMN_2344662	HMGA2	8091	2.36	5.83	7.31	4.61E-04	4.31E-02	0.64
ILMN_1775268	HECW2	57520	2.34	8.28	9.38	1.26E-04	3.88E-02	1.88

Differential expression between paired samples (MSC in CLL co-culture vs. the respective untreated MSC) was analyzed using Illumina probes. The table shows Illumina probe ID, gene symbol, Entrez gene ID (Gene ID), the $\log_2$ fold change (logFC), the average $\log_2$ expression (av. expr.), the moderated test statistic (t), raw *p* value (*p* value), Benjamini-Hochberg adjusted *p* value (adj. *p* value) and the log-odds that the gene is differentially expressed (B). n.a.: not available. Adj. p value < 0.05 (454 probes); the top 50 upregulated probes, ordered by logFC, out of 454 significantly deregulated probes are listed.

Table S5. List of flow cytometry antibodies.

Antigen	Fluorochrome	Clone	Distributor
anti-human CD5	FITC	L17F12	BD biosciences, Heidelberg, Germany
anti-human CD11b	PE	ICRF44	BD biosciences, Heidelberg, Germany
anti-human CD19	PE	HIB19	BD biosciences, Heidelberg, Germany
anti-human CD19	APC	HIB19	BD biosciences, Heidelberg, Germany
anti-human CD20	APC	2H7	BD biosciences, Heidelberg, Germany
anti-human CD44	PE	G44-26	BD biosciences, Heidelberg, Germany
anti-human CD45	PE	HI30	BD biosciences, Heidelberg, Germany
anti-human CD73	APC	AD2	BD biosciences, Heidelberg, Germany
anti-human CD86	PE	n.a.	BD biosciences, Heidelberg, Germany
anti- human CD90	FITC	5E10	BD biosciences, Heidelberg, Germany
anti-human CD105	PerCP-Cy5.5	266	BD biosciences, Heidelberg, Germany
anti-human HLADR	PE	L243	BD biosciences, Heidelberg, Germany
anti-mouse CD3	APC	145-2C11	BioLegend, San Diego, CA, USA
anti-mouse CD3	FITC	145-2C11	BioLegend, San Diego, CA, USA
anti-mouse CD4	AF700	OKT4 or RM4-5	BioLegend, San Diego, CA, USA
anti-mouse CD4	PerCP-Cy5.5	RM4-4	BioLegend, San Diego, CA, USA
anti-mouse CD5	PE	S3-7.3	BioLegend, San Diego, CA, USA
anti-mouse CD5	APC	53-7.3	BioLegend, San Diego, CA, USA
anti-mouse CD8a	APC Cy7	53-6.7	BioLegend, San Diego, CA, USA
anti-mouse CD11b	PerCP-Cy5.5	M1/70	eBioscience/Thermo Fisher Scientific, Waltham, MA, USA
anti-mouse CD19	PE	eBio1D3	eBioscience/Thermo Fisher Scientific, Waltham, MA, USA
anti-mouse CD19	PE Cy7	eBio1D3	eBioscience/Thermo Fisher Scientific, Waltham, MA, USA
anti-mouse CD19	FITC	eBio1D3	eBioscience/Thermo Fisher Scientific, Waltham, MA, USA
anti-mouse CD45	AF700	30-F11	BioLegend, San Diego, CA, USA
anti-mouse CD45	PE-Cy7	104	BioLegend, San Diego, CA, USA
anti-mouse CD45	PerCP Cy5.5	30-F11	BioLegend, San Diego, CA, USA
anti-mouse CXCR1	PE-Dazzle	SA011F11	BioLegend, San Diego, CA, USA

Table S6. List of flow cytometry antibody panels,.

Cells Stained	Description	Antibody Panel (Dilution)
human PBMCs from CLL patient-derived blood	determine CLL cell content	CD5 FITC (1:10), CD19 APC (1:10)
	confirm CLL cell purity after CD19 MACS	CD5 FITC (1:10), CD20 APC (1:10)
human MSCs from healthy donor bone marrow aspirates	analyze the immunophenotype and exclude contamination with hematopoietic cells	panel 1: CD73 APC (1:20), CD90 FITC (1:20), CD105 PerCP Cy5.5 (1:20), CD44 PE (1:10)<br>plus panel 2: CD73 APC (1:20), CD90 FITC (1:20), CD105 PerCP Cy5.5 (1:20), CD45 PE (1:10), CD11b PE (1:10), CD19 PE (1:20), HLADR PE (1:10)
human CLL cells	quantify CD86 surface levels under different conditions	CD86 PE (1:20)
murine blood	determine leukemic cell load after Eμ-*TCL1* adoptive transfer and in controls	panel 1 (refers to Figure S2): CD5 PE (1:100), CD19 FITC (1:200), CD45 PerCP (1:200)<br>panel 2 (refers to Figure 5a): CD5 APC (1:200), CD19 FITC (1:100), CD45 AF700 (1:200)<br>panel 3 (refers to Figure 5c): CD5 APC (1:200), CD19 PE Cy7 (1:200), CD45 PerCP Cy5.5 (1:200)
murine splenic single cell suspensions		panel 1 (refers to Figure S2): CD5 PE (1:100), CD19 FITC (1:200), CD45 PerCP (1:200)<br>panel 2 (refers to Figure 5d): Fixable viability stain 700 (1:1000, (BD biosciences, Heidelberg, Germany), CD5 APC (1:200), CD19 PE Cy7 (1:200), CD45 PerCP Cy5.5 (1:200)
murine peritoneal cavity cells		CD5 PE (1:100), CD19 FITC (1:200), CD45 PerCP (1:200)
murine bone marrow cells	confirm successful T cell depletion in cells used to generate bone marrow chimeric mice	CD45 AF700 (1:200), CD3 APC (1:200), CD4 PerCP Cy5.5 (1:200), CD8 APC Cy7 (1:200),
murine blood	confirm successful bone marrow reconstitution by analysis of leukocytes in peripheral blood in chimeric mice	CD3 FITC (1:200), CD19 PE (1:200), CD4 AF700 (1:200), CD8 APC-Cy7 (1:200), CD11b PerCP-Cy5.5 (1:200), Cx3cr1 PE-Dazzle (1:200), CD45 PE-Cy7 (1:200)

Table S7. List of qPCR primers.

Primer Name	Sequence 5′ to 3′
71 ACTG1 human fwd	CCGAGCCGTGTTTCCTTCC
71 ACTG1 human rev	GCCATGCTCAATGGGGTACT
6347 CCL2 human fwd	AGTCTCTGCCGCCCTTCT
6347 CCL2 human rev	GTGACTGGGGCATTGATTG
1513 CTSK human fwd	GACAGGGGTACTTTGAGTCCA
1513 CTSK human rev	GACAGGGGTACTTTGAGTCCA
2919 CXCL1 human fwd	TCTTGAGTGTGGCTATGACTTCG
2919 CXCL1 human rev	GTGGCCACTGAACTGCGCT
3576 CXCL8 human fwd	TCTCTTGGCAGCCTTCCTGA
3576 CXCL8 human rev	GTGGAAAGGTTTGGAGTATGTCTTTA
2512 FTL human fwd	CAGCCTGGTCAATTTGTACCT
2512 FTL human rev	GCCAATTCGCGGAAGAAGTG
3553 IL1B human fwd	CTGTCCTGCGTGTTGAAAGA
3553 IL1B human rev	TTGGGTAATTTTTGGGATCTACA
9173 IL1RL1 human fwd	GAAAACCTAGTTACACCGTGGAT
9173 IL1RL1 human rev	GCAAACACACGATTTCTTTCCTG
3569 IL6 human fwd	TCCTGATCCAGTTCCTGCAG
3569 IL6 human rev	GCTGCGCAGAATGAGATGAGT
4312 MMP1 human fwd	GGGAGATCATCGGGACAACTC
4312 MMP1 human rev	GGGCCTGGTTGAAAAGCAT
4314 MMP3 human fwd	AGTCTTCCAATCCTACTGTTGCT
4314 MMP3 human rev	TCCCCGTCACCTCCAATCC
6235 RPS29 human fwd	CGCTCTTGTCGTGTCTGTTCA
6235 RPS29 human rev	CCTTCGCGTACTGACGGAAA
6696 SPP1 human fwd	GCCGAGGTGATAGTGTGGTT
6696 SPP1 human rev	TGAGGTGATGTCCTCGTCTG
7431 VIM human fwd	ACACCCTGCAATCTTTCAGACA
7431 VIM human fwd	GATTCCACTTTGCGTTCAAGGT

Primers are named with the official gene symbol, gene ID, species, and fwd (forward) or rev (reverse).

Table S8. List of antibodies used for immunofluorescent stainings of human CLL lymph nodes.

Primary Antibody	Distributor
anti-alpha smooth muscle actin antibody, rabbit	Abcam, Cambridge, United Kingdom (#ab5694)
anti-CD68 antibody, mouse	Abcam, Cambridge, United Kingdom (#ab955)
anti-human progranulin antibody, goat	R&D Systems, Minneapolis, MN, USA (#AF2420)
**Secondary Antibody**	**Distributor**
Alexa fluor 488 donkey anti-goat antibody	Invitrogen/Thermo Fisher Scientific, Waltham, MA, USA (#A11055)
Alexa fluor 488 donkey anti-rabbit antibody	Invitrogen/Thermo Fisher Scientific, Waltham, MA, USA (#A21206)
Cy3 rabbit anti-mouse antibody	Jackson ImmunoResearch Laboratories, West Grove, PA, USA
Cy5 donkey anti-goat antibody	Jackson ImmunoResearch Laboratories, West Grove, PA, USA

The following antibody combinations and dilutions were used: (i) anti-human progranulin antibody (goat, 1:100) – Alexa fluor 488 anti-goat antibody (donkey, 1:300); anti-human CD68 antibody (mouse, 1:200) – Cy3 anti-mouse antibody (rabbit, 1:300) (ii) anti-human progranulin antibody (goat, 1:100) – Cy5 anti-goat antibody (donkey, 1:300); anti-human alpha smooth muscle actin antibody (rabbit, 1:100) – Alexa fluor 488 anti-rabbit antibody (donkey, 1:300).

Supplementary Methods

1. Supplementary cell culture experiments (Figure S1)

BJ fibroblasts were purchased from ATCC, Manassas, VA, USA. C57BL/6 fibroblasts were kindly provided by Dr. Gloria Lutzny and Dr. Ingo Ringshausen, formerly Technical University, Munich, Germany and generated as described previously [2].

BJ fibroblasts or C57BL/6 fibroblasts were either cultured alone or co-cultured with 5×10^5 CLL PBMCs in 250 µL DMEM supplemented with 4.5 g/L glucose, 4 mM L-glutamine, 10 % fetal calf serum, and 1 % Penicillin / Streptomycin (10,000 U/mL) in a 48-well plate format.

2. Supplementary mouse studies (Figure S2)

Total splenocytes of a leukemic transgenic Eµ-*TCL1* mouse (leukemic cell load of 91.1 % $CD5^+$ $CD19^+$ cells out of viable splenocytes) were suspended in PBS. 2×10^7 of those splenocytes/mouse were transferred to $Grn^{+/-}$ mice and $Grn^{-/-}$ mice by i.p. injection. All recipient mice were 7-8 weeks old males and littermates.

Information on mouse lines, authorization of studies, and isolation protocols of blood and spleen are described in the main part of the manuscript. Additional isolation of peritoneal cavity cells was performed as previously described [3].

References

1. Döhner, H.; Stilgenbauer, S.; Benner, A.; Leupolt, E.; Krober, A.; Bullinger, L.; Dohner, K.; Bentz, M.; Lichter, P. Genomic aberrations and survival in chronic lymphocytic leukemia. *The New England journal of medicine* **2000**, *343*, 1910-1916, doi:10.1056/nejm200012283432602.
2. Lutzny, G.; Kocher, T.; Schmidt-Supprian, M.; Rudelius, M.; Klein-Hitpass, L.; Finch, A.J.; Durig, J.; Wagner, M.; Haferlach, C.; Kohlmann, A., et al. Protein kinase c-beta-dependent activation of NF-kappaB in stromal cells is indispensable for the survival of chronic lymphocytic leukemia B cells in vivo. *Cancer cell* **2013**, *23*, 77-92, doi:10.1016/j.ccr.2012.12.003.
3. Ray, A.; Dittel, B.N. Isolation of mouse peritoneal cavity cells. *Journal of visualized experiments : JoVE* **2010**, 10.3791/1488, doi:10.3791/1488.
